# Structured Integration and Alignment Algorithm: A Tool for Personalized Surgical Treatment of Tibial Plateau Fractures

**DOI:** 10.3390/jpm11030190

**Published:** 2021-03-10

**Authors:** Flaviu Moldovan, Adrian Gligor, Tiberiu Bataga

**Affiliations:** 1 IOSUD Doctoral School, “George Emil Palade” University of Medicine, Pharmacy, Science, and Technology of Targu Mures, 540139 Targu Mures, Romania; 2 Biomedical Research Center, “George Emil Palade” University of Medicine, Pharmacy, Science, and Technology of Targu Mures, 540139 Targu Mures, Romania; adrian.gligor@umfst.ro; 3 Department of Orthopedics—Traumatology, “George Emil Palade” University of Medicine, Pharmacy, Science, and Technology of Targu Mures, 540139 Targu Mures, Romania; tbataga@gmail.com

**Keywords:** orthopedic surgery, tibial fracture, personalized treatment, alignment algorithm, workflow, three-dimensional printing

## Abstract

The planning of the surgical treatment in orthopedics, with the help of three-dimensional (3D) technologies, arouses an increasing scientific interest. Scientific literature describes some semi-automatic reconstructive attempts at fragmented bone fractures, but the matching algorithms presented are likely to improve. The aim of this paper is to develop a new method of aligning fragments of comminutive fractures. We have created a structured integration process and an alignment algorithm integrated in a clinical workflow for personalized surgical treatment of fractures. The provided solution is able to align the surfaces of bone fragments derived from the segmentation process of volumetric tomographic data. Positional uncertainties are eliminated interactively by the user, who selects the corresponding pairs of fracture surfaces. The final matching and the right alignment are performed automatically by the innovative alignment algorithm. The paper solves a challenging problem for the reconstruction of fractured bones, namely the choice of the optimal matching option from the situation in which surface portions of a fracture fragment correspond to multiple high fragments. The method is validated in practice for preoperative planning of a 49-year-old male patient who had a tibial plateau fracture of Schatzker type VI.

## 1. Introduction

Serious trauma suffered as a result of vehicle accidents or falls from heights in which a large amount of energy is involved often results in fractures in which bones are multifragmentary. Also, bone density disorders may lead to an increase in the probability of unexpected fractures [1], or the association between bone and muscle tissue reduction with risk factors for some chronic diseases [2]. Even if personalized web-based predictive tools in orthopedic surgery are becoming increasingly available [3], fractures are often difficult to detect and analyze on film scans. Computer Assisted Diagnosis systems based on convolutional neural network are limiting the number of wrong diagnoses [4] and can be used for improving diagnosis accuracy, as demonstrated for proximal femur fractures that is aimed to be extended to all bone segments, and to become a tool commonly used in hospital practice [5].

Injuries resulting in fractures of the joints, such as the knee, can lead to fragmented bones, which require precise surgical reconstruction [6]. Exact bone restoration allows the reconstruction of the axes, being a critical factor in determining the favorable clinical prognosis [7].

Severe joint fractures occur most frequently in young adult patients, and the objectives of treatment are to stabilize the patient and achieve a bone union as quickly as possible, in a position of alignment of the limbs, and avoid posttraumatic osteoarthritis. This is a serious health condition comparable to other major disorders, such as stroke, heart disease or diabetes. Its effect is a decrease in general health, loss of work capacity, or even disability [8]. Patients may require reconstructive surgery, with multiple interventions over a longer period of time, to maintain the joint function or even the joint replacement.

The scientific literature on semi-automatic or fully automatic reconstruction of bone fragments is quite scarce, with research mainly on medical imaging, bone segmentation and surface recording [9]. Recent advances in augmented reality real-time solutions for robotic surgery navigation with modular approach during invivo robotic surgery and segmentation of the whole procedure in a set of stages allow for a very good tracking strategy for fracture reconstruction [10].

Research on bone reconstruction is generally limited to simple fractures from a small number of fragments [11]. The recent emergence of 3D technologies has increased the potential to investigate personalized treatment of fractures [12,13], to propose preoperative plans [14], but also to develop customized treatment solutions in cases of simple fractures [15] or complex ones [16,17], specific instrumentation, osteointegrated implants for traumatology and orthopedics [18]. This allows for improved accuracy and efficiency of orthopedic surgeries for internal fixation [16].

By applying classical methods of open reduction, bone fragments can be devascularized at the same time as soft tissue is compromised. This requires minimally invasive personalized orthopedic surgery that treats severe comminuted fractures and allows for satisfactory results [19].

Given the spectacular evolution of 3D printing technologies, it is necessary to transpose them into clinical workflows. Orthopedic surgeons must specialize in the latest technological developments in order to integrate applications in personalized patient treatments into clinical practice [20].

This paper presents a system of semi-automatic virtual reconstruction of bone fractures from volumetric computed tomography (CT) data. We extended numerical techniques developed in the field of vision and computer graphics for the reconstruction of broken archaeological fragments [21,22,23] and applied them to the reconstruction of highly fragmented bone fractures. While some of these techniques are directly applicable to bone reconstruction, there are many new challenges [24].

Volumetric CT data from clinical applications usually have a resolution of about 0.2–0.5 mm in the acquisition plane and slightly larger (0.3–0.5 mm) in the direction in which the images are acquired. CT scan data reflect an average tissue density per volume/thickness of the finished slice, relative to the associated partial volume. Because the segmented surfaces in the CT data show great variability, extracting the desired surfaces from the CT volumetric data is a difficult problem in the segmentation process.

On the other hand, the noise is not stationary, and its structure is dependent on the architecture of the segmentation algorithm used for surface extraction, which is usually much more intense in difficult segmentation regions.

In the process of generating fractures, when the living bone is subjected to a high-energy trauma, it tends to split, generating parts that usually have a single fracture surface that varies slightly and corresponds to one, two or more fragments. In comminuted fractures, two or more such surfaces can be identified, which have more associated fragments. The fragments show regions of smooth surfaces which, during reconstruction, fit both the large basic fragment of bone from which they originate and also other smaller fragments.

Consequently, the bone reconstruction algorithm must allow the fracture surfaces to facilitate a partial match with another fracture surface from another bone fragment, and which in turn may partially match a third fragment.

In bone reconstruction, the surface matching step excludes the application of perfect one-to-one matching techniques because the variability of the segmentation can lead to attempts to match fragments already aligned elsewhere, or interpenetrating surfaces, or even open surfaces created by empty volumes.

The objective of the research is to develop a general mathematical model and software solution which is an algorithm for structured integration and alignment of the comminuted fracture surfaces. Its application should reduce the intervention of the human operator and optimize the matching process.

## 2. Materials and Methods

### 2.1. Virtual Reconstruction of Fractures

Obtaining a precise restoration of the initially unfractured bone from its fragments is a critical factor in determining the clinical prognosis, especially in cases of comminuted tibial plateau fractures. Personalized treatment requires the development of algorithms for structured integration and alignment of comminuted fracture surfaces.

**Figure 1:**
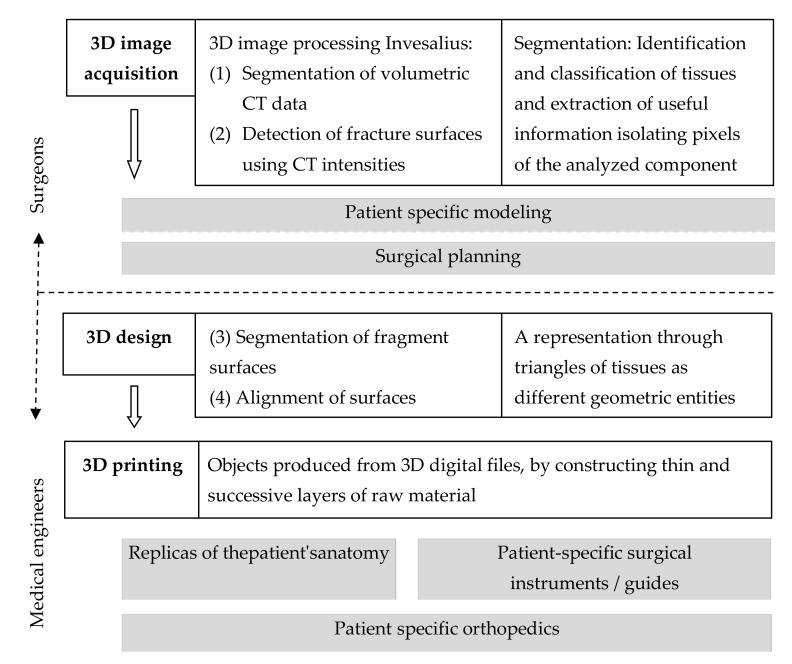
Clinical workflow for structured integration process supported by an alignment algorithm for personalized surgical treatment of fractures.

We created a clinical workflow for structured integration process supported by an alignment algorithm for personalized surgical treatment of fractures which integrates 3D technologies (Figure 1).

The method of fracture virtual reconstruction using volumetric CT data was structured in four steps:

- Step 1: Segmentation of volumetric CT data—aimed to generate closed discrete surfaces, i.e., surfaces of bone fragments which can be aligned later.

**Figure 2:**
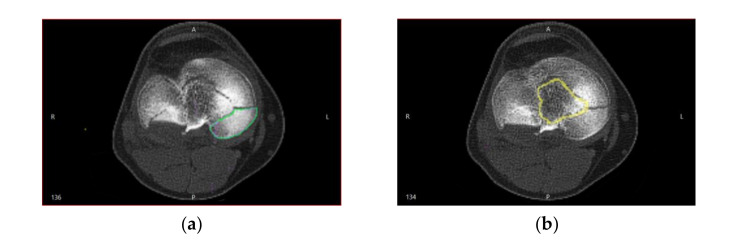
Comminutive tibial fractures shown in CT sections used in the process of the bone fragment fracture surfaces identification. The bone fragment segmented surface is delimited in color: (**a**) the inside of the bone has a higher intensity than that of the cortex; (**b**) the estimated surface segmentation is at a distance from the cortical surface.

The segmentation algorithm was not linear, which complicated the relationship between the noise distribution identified in CT by Hounsfield intensities and the noise existing in the spatial positions of the locations of the segmented surfaces. Therefore, the positions of the points in the segmented CT images were interpreted by real geometric surface noises, in which each point had a unique and unknown noise distribution, and estimating the location of the surfaces of bone fragment fractures was a challenge (Figure 2).

- Step 2: Detection of fracture surfaces using CT intensities—a surface covering was generated for each segmented bone fragment.

The technique used to identify the pairs of points on the segmented surfaces corresponding to the fracture surfaces (i.e., the surface regions generated when the bone fragments are separated) required that each point on the network surface be assigned a Hounsfield intensity that was known from the process of segmenting the bone surfaces in the previous step.

Because the density of cortical bone tissue differs significantly from cancellous bone tissue, the Hounsfield intensity on a given bone surface helped to shape the intact surfaces of the fracture. One finding was that the vast majority of fractured surfaces in the metaphyseal region (near the joint surface) originated in lower density bone. The tibia and long bones generally consisted of cortical bone tissue on the outer surface and cancellous bone tissue inside the bone. Cortical bone tissue is very dense, usually having CT intensities commonly located in the rage 700–2500 Hounsfield units. This tissue is the thickest along the middle bone axis and becomes very thin in the metaphyseal regions near the joints. The cancellous tissue has a variable density, usually having CT intensities around 700 Hounsfield units. 

Because cancellous bone tissue usually has substantially lower Hounsfield intensities compared to cortical bone tissue and is located exclusively within the bone, to distinguish between points on the intact surface and points on the fractured surface, we used the information provided by the Hounsfield intensities in the vicinity of the points on the segmented surfaces.

**Figure 3:**
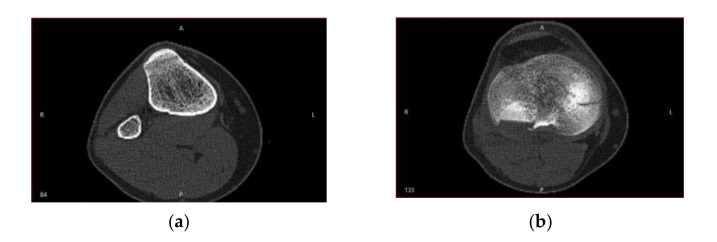
Cortical and cancellous tissue view in a CT: (**a**) high-intensity circular regions show thick cortical bone tissue in the median area of the tibia; (**b**) more proximal, there is much less contrast between the regions of cortical and cancellous bone tissue.

Cortical bone tissue became very thin and less dense in the distal and proximal regions of the bones. Figure 3 shows the CT sections that illustrate this behavior. 

Along with the high-intensity circular regions that had thick cortical bone tissue (Figure 3a), situations may have arisen when following the segmentation, the analyzed surface was located at a distance from the cortical bone surface (Figure 3b), which made it challenging to identify the fracture surfaces on the segmented bone surfaces. This problem was addressed by using binary operations between multiple 3D masks in order to estimate with high accuracy the true bone surfaces, based on Hounsfield intensities. To minimize errors, we considered a lower bound threshold.

- Step 3: Segmentation of fragment surfaces—with the help of Hounsfield intensities, the surfaces were segmented into intact surfaces and fracture surfaces.

We used intensities and the adopted threshold from previous step in order to minimize errors. This allowed the segmentation of surfaces into intact surfaces and fracture surfaces. In this way, we obtained probable pairs of surfaces, but which did not match exactly.

- Step 4: Alignment of surfaces—given the lack of a reliable system for direct correspondence of fractured surfaces, the intervention of the operator was required.

Using these pairs of fracture surfaces, a multi-body alignment scheme was applied, which used a modified method of the Iterative Closest Point (ICP) algorithm to align the pairs of fracture surfaces.

To do this, we started from an approximate initial alignment, and the ICP algorithm pairs two points, the closest on two surfaces (which were described by networks of points arranged in triangular meshes which formed a net). Then by 3D movements, the pairs of points on the two surfaces were better aligned, and these steps were further iterated as long as the alignment was improved.

The ICP method can provide a measure of confidence that a perceived surface (e.g., a fracture fragment) belongs to a reference surface (e.g., an entire bone that is fractured), during a process called matching. In this case, a local match was used, as the perceived area corresponded only to a certain region of the reference area. In principle, a matching process requires six degrees of freedom: three for rotation and three for translation, and these were determined using correspondence of the nearest neighbor type.

### 2.2. Matching and Alignment Methods

There are two basic methods for identifying the matches between the points on the dislocated surface of the bone fragment and the points on the basic surface of the bone, in which both surfaces are described by networks of points arranged in triangular meshes, namely: a) finding the closest point, and b) the extension of the normal vector to the point on the deployed network until it intersects the other base network [25]. Accordingly, the two major methods of alignment can be used to align points and surfaces: (a) point-to-point method and (b) point-to-plane method.

**Figure 4:**
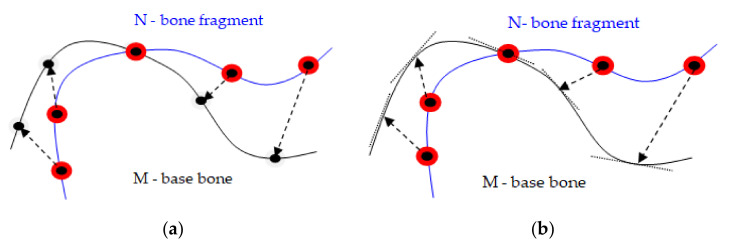
(**a**) Point-to-point method: points are matched to discrete points; (**b**) Point-to-plane method: points are suitable for continuous tangent planes.

The point-to-plane method is preferable to the point-to-point method for the alignment in pairs of two bone fragments modeled by volumetric scans of the bone fragments. This method converges faster in order of magnitude. As the surfaces get relatively close one to the other, most points in or near the overlapping area can find a compatible pair nearby, even if it is necessary for one of the surfaces to slide along the other for better alignment. In the point-to-point method, Figure 4a, each point of the point cloud describing the dislocated surface of the bone fragment N was associated with a discrete approximation of the other base surface of the bone M and even if a few pairs of points on the two surfaces were ideally paired, they opposed a wider displacement of surfaces that would lead to better alignment. 

In contrast, in the point-to-plane method, Figure 4b, each point was associated with a continuous linear approximation of the pair surface, and the pairs of false points did not obstruct the alignment process, because each point could slide on a tangent plane on the surface of its pair. It better describes the process of matching bone fragments, in which there was some elasticity and allowed even a faster implementation of the programming algorithm.

For the reasons presented, the method of the nearest iterative point [26] could not be used in the reconstruction of fractures, because there had to be a perfect correspondence between the points of the two surfaces, a requirement that can rarely be met in databases. For these reasons we developed a heuristic method, which had the following steps:

1. For each point on the N network (fracture fragment) the nearest position on the surface M (base bone) was sought. The identified point could be a point in the network node, it could be a point inside a triangle, or it could be on the side of a triangle. Allowing a match on the continuous surface M versus matching with the network points increased the degree/accuracy of matching between the two surfaces;
2. The pairs of points which are too far apart were dropped;
3. The multiple pairs of points which were made with a boundary of the core bone network were eliminated;
4. There was a rigid transformation which minimized the square of the distance between the pairs of points;
5. The procedure was iterated until convergence was achieved;
6. The algorithm was applied on an increasingly hierarchically detailed network.

### 2.3. Constraints Applied to the ICP Algorithm

The constrained ICP algorithm differed from the classical ICP algorithm by the following:

1. We added a distance threshold to the method based on the nearest iterative point to avoid matching any point N on the network with other points removed from the surface M that probably did not correspond to N;
2. Limiting the distance between the corresponding pairs of points allowed one to perform step 2, removing pairs at a distance, while searching for the closest points in step 1.
  Finding the nearest points could be much advanced if a subdivision of the space was created in which according to the distance threshold the network peaks were placed. Because the dimensions of the triangles had a limited value set at the stage of creating the meshes, all triangles up to a certain set distance could be searched without the risk of losing any part of any triangle. This way of searching for the nearest point was a query that we defined as such:

(1) $$Query\_routine\left(N,d,M\right)\;\begin{array}{l} 1—identifies\;the\;nearest\;point\;on\;the\;M\;network\;to\;the\;N\;point\\ 0—does\;not\;identify\;anything\;if\;there\;is\;no\;point\;on\;the\;network\;M\;at\;distance\;d \end{array}$$

**Figure 5:**
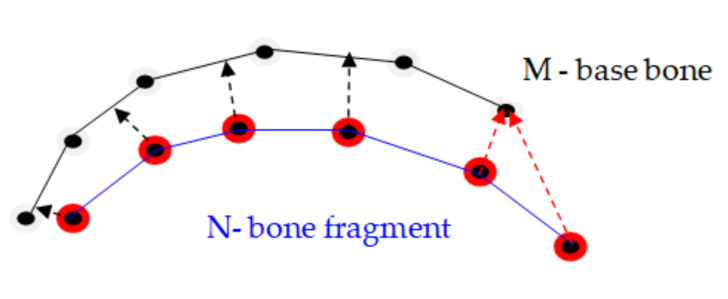
Matching the points between the N and M nodes of two surfaces (defined by the meshes of triangular networks). Vectors in red indicate matches that should be avoided, as they will cause the N surface to be erroneously shifted up and to the left.

3. A limitation was included for the delimitation points which were not allowed to be part of the surfaces matching process. These points were located on a triangle marginal side that was not common to the side of another triangle. Figure 5 illustrates how such a match could move a surface in the opposite direction to most point correspondences.

The essence of the closest iterative point algorithm is to find a rigid transformation that minimizes the square of the distance between the pairs of points.

As we have shown above, not all intervals in which the points are located have the same error limits in determining their position. For this reason, an optional weighting term for minimizing errors was also included, which evaluated the positioning uncertainties in the surface matching process [27]. This term was called trust and indicated the extent to which we were certain about the position of a particular point:

(2) $$\mathrm{Trust}=w_{i}\;=\;\begin{array}{l} 1—the\;operator\;is\;certain\;about\;the\;the\;position\;of\;the\;point\\ 0—the\;operator\;is\;not\;certain\;about\;the\;position\;of\;the\;point \end{array}$$

The confidence of a point M located on the node of a network that wraps a surface is the scalar product of the surface normal at point M and the vector formed between point M and a light source that scans the geometry of the surface during the segmentation process. The surface normal at point M means the average of the norms of the triangles encountered at point M. The solution now is to find a minimum of weighted square distances:

(3) $$Min\varepsilon \;=\sum_{i=1}^{n}w_{i}\{\left[M_{i}\right]-\left[R\right]\left(\left[N_{i}\right]-[N_{c}\right])-\left[T\right]{\}}^{2}$$

where $\left[M_{i}\right]$ and $\left[N_{i}\right]$ are the pairs of points in the space, while $\left[N_{c}\right]$ represent the $\left[N_{i}\right]$ point centroid. Translation vector $\left[T\right]$ is the difference between the two centroid of the points $\left[M_{i}\right]$ and $\left[N_{i}\right]$ respectively.

### 2.4. Matching Surfaces in Practice

The matching method described in the previous paragraph can be accelerated by matching more and more detailed meshes (triangles) in a hierarchy. A network mesh hierarchy is usually used. Each of the meshes uses a single number of interval points used in the higher hierarchical level. Sub sampling allows the construction of lowercase meshes that are part of the hierarchy. The process starts by running the ICP at the lowest network level, after which the next level in the hierarchy uses the transformation obtained as the initial configuration. At each advance in the hierarchy the distance of the matching threshold d is reduced by half.

Combining multiple surfaces into a single prototype is the major problem of matching surfaces. The purpose of matching is to obtain representations of the fracture fragments that correspond to the anatomy of the bone.

This section examines how two triangular meshes belonging to two bone fragments can be mated in an individual surface that allows anatomical reconstruction. The complete topology of the bone was made by matching the triangles one by one, thus obtaining a final triangular network of the two overlapping surfaces.

Thus, the matching of the two triangular stitches was done by three iterations:

#### 2.4.1. Removal of Redundant Surfaces

Before joining two triangular grids, some triangles were removed until the two grids overlapped. In this iteration, those triangles that were in a sense “redundant” were removed from each network because the opposite network comprised an uninterrupted mesh in the same spatial position. Even if the iteration eliminated some triangles on the networks, data were not deleted, as all points in those intervals would eventually be used to find consensus geometry.

Two triangular networks M and N were considered, and the process of removing their redundant portions consisted of repeating the following two instructions, until both networks remained unchanged:

(a) The redundant triangles were removed from the surface of the network N;
(b) The redundant triangles on the surface of the network M were eliminated.

Redundant triangles were removed from the N network by querying the M network. This used the interrogative routine (1).

The nearest positions on the M network of the triangle peaks were searched. A triangle was considered redundant if the three queries performed within the tolerance distance d identified only points of the network M that were not on the edge.

#### 2.4.2. Network Matching

The removal of triangles described above facilitated the easy overlap of two networks.

**Figure 6:**
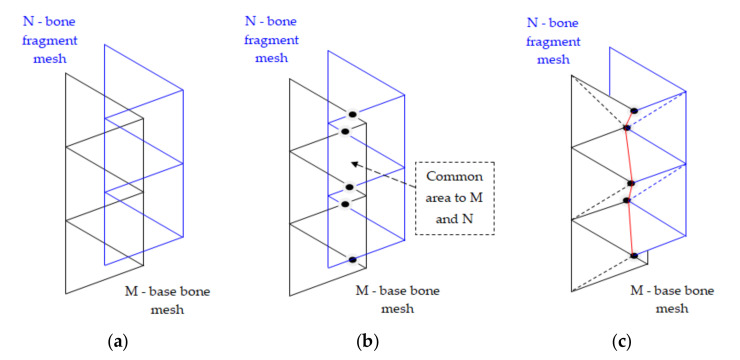
The networks M and N are cut at the common boundary: (**a**) the intersection between the edges of the M and N meshes allows determination of triangle intersection points; (**b**) portions of triangles on the M and N meshes are removed; (**c**) both networks are limited at the points of intersection and new triangles are formed.

Figure 6a shows two overlapping networks, and Figure 6c shows the result of the overlap by cutting some portions of the triangles.

We shall examine in more detail the process of elimination (cutting) triangles on meshes and suppose we act on two networks that are in a common plan, Figure 6a. To cut from the two grids M and N, we first had to determine the common points of intersection of the triangles sides that form the two grids, Figure 6b. These points were joined with a red line in Figure 6c, after which the areas common to the two networks were removed and new triangles were constructed up to the contact line, which are marked with dashed lines. The vertices of the new triangle generated were determined and recorded using a triangulation routine containing all the vertices of the newly formed network. This grid matching iteration was extended into three-dimensional space by accurately identifying all the intersection points.

Another problem is that in three-dimensional space, the edges of the M and N networks do not intersect, they are usually staggered.

**Figure 7:**
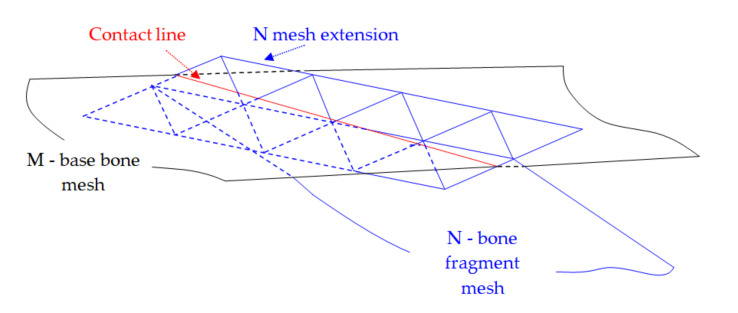
Extended network boundary for cutting in three-dimensional space.

To correct this, the limit of the N network was extended by an “additional” vertical wall. In essence, a wall was created that extended around the boundary of the N network and was approximately perpendicular to the entire boundary of the N network. As shown in Figure 7, the mesh extension consisted of a group of triangles that formed the wall. Finding intersection points with the edges of the M network, we needed to find the points through which these boundaries cross the walls of the triangles. These intersection points were then moved to the nearest positions on the edge of the proper wall. Otherwise, the matching process continued as described above.

#### 2.4.3. Remove Small Triangles Inserted during Matching

The network matching process can introduce small arbitrary triangles into the networks. For the small triangles it is used the routine for deleting triangles.

If a node in the network falls under a threshold specified by the user, that node and all triangles that have that node at its peak are deleted. Preferably triangle points are deleted that were introduced as new nodes during the network matching process.

## 3. Results

**Figure 8:**
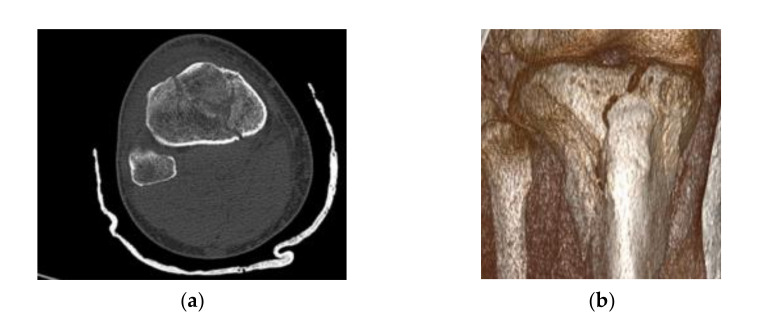
(**a**) Digital Imaging and Communications in Medicine (DICOM) image; and (**b**) RadiAnt DICOM image of the patient included in the study.

In order to provide an estimation of the execution times on our system by employing the Invesalius [28], MeshLab [29] and FreeCAD software [30] we tested our model for the alignment process in a 3D reconstruction process for a right leg Schatzker VI type fracture of a 49 year old male (Figure 8).

**Figure 9:**
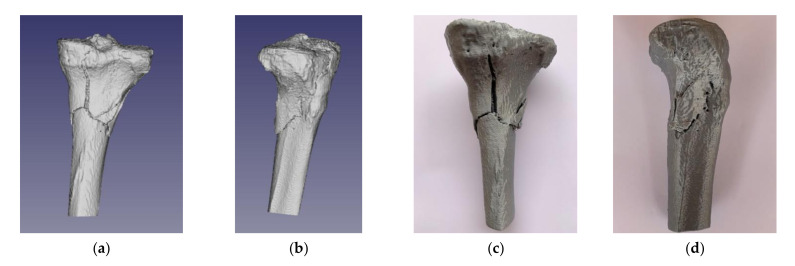
Tibial plateau fracture Schatzker type VI: (**a**) posterior view of the 3D segmented model; (**b**) lateral view of the 3D segmented model; (**c**) posterior view of the 3D printed replica; (**d**) lateral view of the 3D printed replica.

Figure 9a,b shows the case of the tibial plateau fracture test in standard triangle language (STL) format, which was prepared for 3D printing. The nature of bone fragmentation resulted in the identification of bone fragments partially detached from the basal bone, with sufficient volume (size) to be considered of clinical importance. Figure 9c,d show the 3D printed replica of the segmented model.

**Figure 10:**
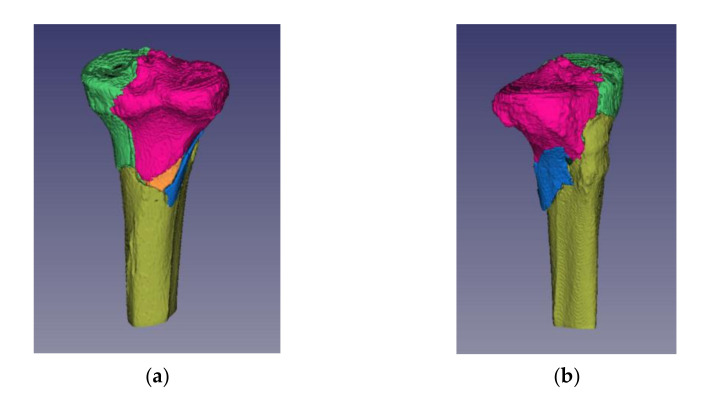
Tibial plateau fracture Schatzker type VI—identification of independent fracture fragments by staining of distinct fracture fragments: (**a**) posterior view; (**b**) lateral view.

Invesalius allows the staining of distinct fracture fragments. This feature by the chosen color fulfilled two important functions (Figure 10):

(1) Acted as a visual aid for the observer in identifying independent fragments, and
(2) Allowed the system to distinguish between the segments that the operator had delimited as separate fracture fragments.

**Figure 11:**
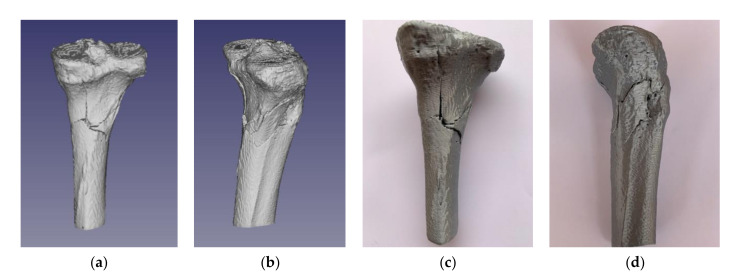
Tibial plateau fracture reconstruction result supported by the alignment algorithm: (**a**) posterior view of the standard triangle language (STL) model; (**b**) lateral view of the STL model; (**c**) posterior view of the 3D printed replica; (**d**) posterior view of the 3D printed replica.

Employment of the alignment algorithm allowed the reduction of bone fragments, which took about 4 min of user interaction and only a few seconds of reconstruction alignment processing time. The reconstruction obtained was excellent, as it was easy to judge by its visual appearance, as demonstrated in Figure 11a,b for the STL model and Figure 11c,d for the 3D printed replica.

**Figure 12:**
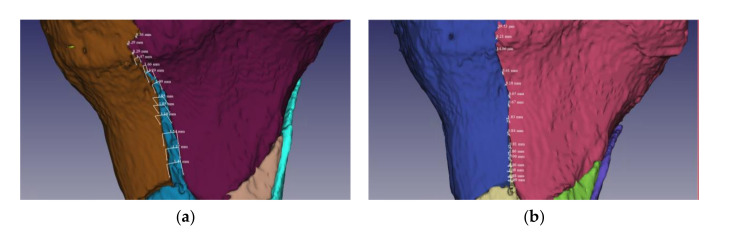
Evaluation of the tibial plateau fracture reduction process by measuring the distances between the fracture fragments and the base bone: (**a**) initial fracture before reduction; (**b**) fracture after reduction supported by the alignment algorithm.

The proposed methodology was evaluated through practical experimental validation by measuring the distances between the fracture fragments and the base bone, on the line of the entire fracture. The measurements were performed before and after the fracture reduction supported by the alignment algorithm, followed by an assessment of the degree of reduction of fracture fragments. Figure 12a,b illustrate the data collected from measurements on a fracture fragment.

**Figure 13:**
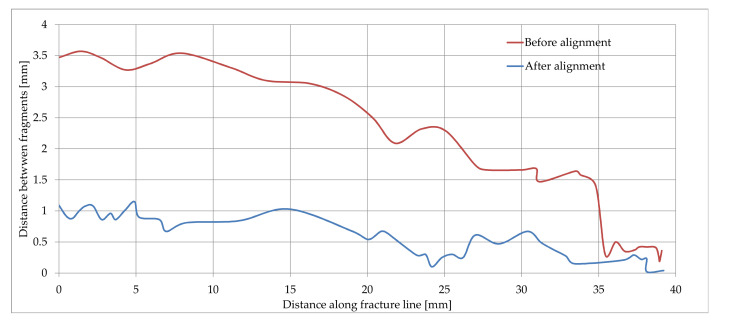
Variation of distances between bone fragments along the fracture line before (curve in red) and after alignment (curve in blue).

Considering the case illustrated in Figure 12, the data samples collected from distances measurements along the fracture line are represented in Figure 13. From the analysis of the two curves representing the variation of the distances between the bone fragments on the initial fracture and after reconstruction, we calculated a degree of alignment between 68.86% for the beginning of the fragment line in which the distances between the bone fragments were reduced from 3.5 mm to 1.09 mm, respectively 100% for the fragment line at 37 mm in which the distances between bone fragments were reduced from 0.4 mm to 0 mm.

This assessment was demonstrated considering one of the severe fracture lines, noticing that the exact match on the entire line was no longer possible due to the damage on the fracture surfaces of the fragments, resulting in complex, irregular contours. The in-depth exploration of the paired surfaces at 2–3 mm, in the immediate vicinity of the fracture line, revealed a high fit, which confirmed a good convergence of the alignment algorithm.

**Figure 14:**
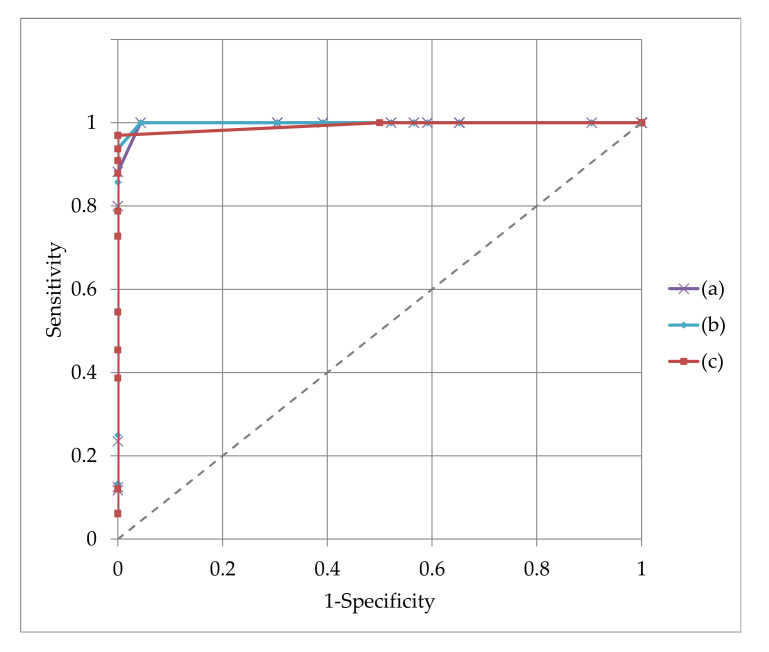
Assessment of the alignment method performance for three values of the evaluation criteria, the mean distance between fragments bones: (**a**) 0.5 mm, (**b**) 0.7 m and (**c**) 1 mm.

In order to get an overview of the performance of the alignment algorithm, the experimental data were evaluated considering different criteria of classifying the quality of the alignment, the considered attribute being the mean of the distance between aligned bones fragments. Considering the measured data samples, the classification of the obtained results for different thresholds in the range of 0.1–3 mm is illustrated in Figure 14. The areas under the receiver operating characteristic (ROC) curves proved that for any of 0.5 mm, 0.7 mm or 1 mm attribute the alignment was performed correctly.

## 4. Discussion

### 4.1. Failure Modes

There are two failure situations of the alignment algorithm. First, it is possible that the collection of radiographic information may be impaired due to the patient’s movement during scanning or incorrect calibration of the device. Consequently, it is not possible to identify any rigid transformation system that aligns all bone fragments simultaneously, due to their registration in different relative landmarks. An alignment of fragments relative to different relative landmarks may be the solution to this undesirable situation. The second failure situation could occur when the paired surfaces are perfectly flat or circular, which would lead to a multitude of alignment possibilities. To control this situation, we have introduced some diagnostic tools in the algorithm, which allow the location of false surfaces and allow process control to avoid accidental slipping to regions without features or atypical ones.

### 4.2. Diagnostic Tools

The alignment is usually inspected by the operator visually and any misalignments can be identified. The distance from one fragment to another may be studied, without this being the most relevant indication on fragment fitting, as there may be regions without well-identified features. In this situation, if the algorithm does not detect a clear alignment, the operator must redefine the numerical features of the misaligned surfaces. The entire fragment alignment process is resumed, while paying special attention to avoid recurrence of the previous situation.

### 4.3. Alignment Workflow

When handling large volumes of data related to several fracture fragments, individual alignment of the fragment pairs is not recommended. It is preferable to have a rough initial alignment, and create first pair records and try to automatically align each pair of fragments that are found to have common surfaces.

## 5. Conclusions

Originality: This structured integration algorithm retains the highest possible connectivity of the triangles created as grid meshes that describe the surfaces in the original images, prior to processing for matching. Integration is accelerated because the process evaluates one-dimensional portions of the network instead of two-dimensional surfaces.

In essence, the process consists of mediating and integrating two geometries, which belong to the fracture fragment and the base bone, in which: (1) the common points on the two surfaces are joined, after which (2) for the points that are not paired, geometry mediation is applied.

The planar point matching process used and the modified ICP algorithm describe well the process of matching the bone fragments, in which there is some elasticity which allows a quick implementation of the programming algorithm.

In conclusion, the proposed method is innovative in that it first identifies the maximum connectivity between the two networks that describe the bone fragment and the base bone, after which it determines the final geometry of the joined surfaces. In this way, the points that easily identify a correspondent are eliminated in the matching process, after which the matching is continued with a limited number of points.

The study limitations occur because the method was tested on a Schatzker type VI fracture. In the case of even more complex fractures the algorithm may require some improvements to identify all small fragments in order to fit.

Another limitation of the study is that physical testing was performed on a virtual model. In vivo, the biological conditions are slightly different; for example, the blood flow may influence the identification of corresponding points on meshes in order to achieve an optimal fit.

Further research: The method is exemplified in the case of tibial plateau fracture but can also be used in other complex orthopedic trauma.

## Data Availability

The data presented in this study are available on request from the corresponding author.
